# NEUROMUSCULAR FITNESS IN EARLY LIFE AND ITS IMPACT ON BONE HEALTH IN ADULTHOOD: A SYSTEMATIC REVIEW

**DOI:** 10.1590/1984-0462/2020/38/2019119

**Published:** 2020-03-09

**Authors:** Cynthia Correa Lopes Barbosa, Catiana Leila Possamai Romanzini, Mariana Biagi Batista, Rômulo Araújo Fernandes, Marcelo Romanzini, Han Kemper, Manuel João Coelho-e-Silva, Enio Ricardo Vaz Ronque

**Affiliations:** a Universidade Tecnológica Federal do Paraná, Campus Apucarana, PR, Brazil.; b Laboratório de Atividade Física e Saúde, Universidade Estadual de Londrina, PR, Brazil.; c Universidade Federal de Mato Grosso do Sul, MS, Brazil.; d Universidade Estadual Paulista “Júlio de Mesquita Filho”, SP, Brazil.; e Amsterdam Public Health Research Institute, Amsterdam, The Netherlands.; f CIDAF (uid/dtp/04213/2019), Universidade de Coimbra, Coimbra, Portugal.

**Keywords:** Muscle strength, Bone mineral density, Childhood, Adolescence, Força muscular, Densidade óssea, Infância, Adolescência

## Abstract

**Objective:**

To systematically review the literature to verify the relationship between neuromuscular fitness indicators in childhood/adolescence and bone strength variables in adulthood.

**Data sources:**

A systematic review was conducted in PUBMED, SCOPUS, SPORTDiscus, Web of Science, PsycINFO, LILACS, and SciELO, covering the entire period until March 2019.

**Data synthesis:**

The search identified 1149 studies. After duplicity analysis and eligibility criteria, four studies were reported. In one study, baseline was childhood and, in the others, adolescence. In childhood, when adjusting the model for age and body mass index, a statistically significant relation was found for girls: standing long jump with quantitative ultrasound index (β=0.11; p<0.05) and with speed of sound (β=0.14; p<0.01). However, when controlling muscular performance in adulthood, the relationship was no longer significant. In adolescence, coefficients ranged from 0.16 for neuromotor battery and bone mineral density (BMD) in the lumbar region to 0.38 for hanging leg lift test and BMD of arms. The explained variance varied between 2% (bent arm hang for BMD total) and 12% (hanging leg-lift for BMD arms), therefore, a higher performance in neuromuscular fitness in adolescence was associated with better bone strength in adulthood.

**Conclusions:**

In adults, bone strength variables showed significant correlation from low to moderate magnitude with neuromuscular fitness indicators in adolescence, but not in childhood, after controlling for adult performance in neuromuscular fitness. However, there is limited evidence to support the neuromuscular fitness in early life as a determinant of bone strength in adulthood.

## RESUMO

**Objetivo:**

Revisar sistematicamente a literatura para verificar a relação entre indicadores da aptidão neuromuscular na infância/adolescência e variáveis de resistência óssea na idade adulta.

**Fonte:**

**de dados:** Revisão sistemática com pesquisa de artigos conduzida no PUBMED, SCOPUS, SPORTDiscus, Web of Science, PsycINFO, LILACS and SciELO, abrangendo todo o período da base até março de 2019.

**Síntese:**

**dos dados:** A busca identificou 1.149 estudos, após análise de duplicidade e dos critérios de elegibilidade, quatro estudos foram relatados. Em um dos estudos, o *baseline* foi a infância e, nos demais, a adolescência. Na infância, ao ajustar o modelo por idade e índice de massa corporal, foi encontrada relação estatisticamente significativa para as meninas: salto em distância com índice quantitativo de ultrassom (β=0,11; p<0,05) e com velocidade do som (β=0,14; p<0,01). Entretanto, ao se fazer o controle do desempenho muscular na idade adulta, a relação deixou de ser significativa. Na adolescência, coeficientes variaram de 0,16 para bateria neuromotora e densidade mineral óssea (DMO) lombar a 0,38 para o teste de elevação de pernas em suspensão e a DMO dos braços. Variação explicada entre 2% (suspensão na barra e DMO do corpo total) e 12% (elevação de pernas em suspensão e DMO dos braços), portanto, melhor desempenho na aptidão neuromuscular na adolescência, mais resistência óssea na idade adulta.

**Conclusões:**

Em adultos, variáveis de resistência óssea apresentaram correlação significativa em magnitude baixa a moderada com indicadores da aptidão neuromuscular na adolescência, mas não na infância, após controlar pelo desempenho adulto na aptidão neuromuscular. No entanto, existe evidência limitada para apoiar a aptidão neuromuscular na juventude como fator determinante da resistência óssea na fase adulta.

## INTRODUCTION

Osteoporosis is characterized by compromised bone strength that predisposes a person to an increased risk of bone fracture.^1^ ^,^ ^2^ Occurring mainly in the hip, vertebrae, and forearm, the fractures negatively influence the quality of life of affected individuals,^3^ leading to dramatic increment health costs and thus being identified as a significant public health concern.^4^

The fragility of bones in senile osteoporosis results from the processes of low peak bone mass and/or bone loss accentuated with advancing age, especially among women.^5^ ^,^ ^6^ Part of the bone fragility is established in early phases of lifecycle and optimizing peak bone mass could mitigate the consequences.^7^ Peak bone mass is the amount of bone mass acquired up to reaching a plateau, which usually occurs until the beginning of the third decade of life.^8^ During this bone mass acquisition period (notably during childhood and adolescence), heredity, sex, hormones, nutrition, and mechanical loads (physical activities) are consensually important determinants of peak bone mass.^5^ ^,^ ^6^

Physical activity consistently affects the development of peak bone mass.^6^ Mechanostat, mechanosensation, and transduction theories are often cited to explain on bone-muscle interaction via muscle contraction.^9^ ^,^ ^10^ ^,^ ^11^ Analysis of the bone-muscle unit during the period of maximum growth shows that muscle development precedes bone development,^12^ ^,^ ^13^ and even considering the genetic determination in this process, muscle strength could be important in bone modeling and strength. Therefore, neuromuscular fitness may be a determinant of the peak bone mass.

Several cross-sectional studies investigated the relationship between neuromuscular fitness indicators and bone strength variables and a positive relation was systematically observed with a magnitude effect that ranges from moderate to large.^14^ ^,^ ^15^ ^,^ ^16^ However, no statistical significance or a lower magnitude of a relationship was observed in longitudinal studies that examined neuromuscular fitness in young people in relation to bone health in adulthood.^17^ ^,^ ^18^ In addition, there are more cross-sectional studies rather than longitudinal studies and a great diversity of methods are used to obtain neuromuscular fitness and bone health indicators.

Research is needed to explain the specific and independent contribution of neuromuscular fitness on the variables of bone strength.^19^ The relationship between indicators of neuromuscular fitness (strength, endurance, and power) and bone health variables (bone mineral density [BMD] and bone mineral content [BMC]) could vary regarding the magnitude, mediating factors and variation over time. Besides, knowing aspects of this relationship, especially during the years of peak bone mass acquisition and stabilization, may be relevant to understand the effects of mechanical stress on the bone matrix and, therefore, to verify if the neuromuscular fitness plays a determinant role in the peak of bone mass.

Given the need to gather available information on the subject, the objective of this study was to systematically review the literature to verify the relationship between indicators of neuromuscular fitness in childhood and/or adolescence and variables of bone strength in adulthood.

## METHOD

This study was carried out following the recommendations of the *Preferred Reporting Items for Systematic Reviews and Meta-Analyses* (PRISMA).^20^ The following databases were searched: Medline/PubMed, SCOPUS, SPORTDiscus, Web of Science, PsycINFO, LILACS, and SciELO. The search covered the entire period of existence of the databases until March 19, 2019, and the only filter used was the language to select studies, English, Spanish, and Portuguese.

The search comprised the following keywords and Boolean operators: (“muscle strength” OR “strength muscle” OR “muscular strength” OR “hand strength” OR “handgrip” OR “grip strength” OR “muscle strength dynamometer” OR “muscle strength dynamometers” OR “physical capacity” OR “physical fitness” OR fitness OR “muscular fitness” OR “muscle endurance” OR “muscle power” OR “explosive strength” OR “muscle fitness” OR “musculoskeletal fitness” OR “motor fitness” OR “neuromotor fitness” OR “motor performance” OR “motor tests” OR “standing long jump” OR “standing broad jump” OR “vertical jump” OR “sit-ups” OR “isokinetic dynamometry” OR “isokinetic”) AND (child OR children OR childhood OR students OR student OR scholars OR scholar OR adolescent OR adolescents OR adolescence OR teen OR teens OR teenager OR teenagers OR youth OR youths) AND (“bone density” OR “bone densities” OR “bone mineral density” OR “bone mineral densities” OR “bone mineral content” OR “bone mineral contents” OR “bone densitometry” OR “bone health” OR “bone strength” OR “bone mass” OR “peak bone mass”) AND (“young adult” OR “young adults” OR “young adulthood” OR “university students” OR adult OR adults OR adulthood).

In the specific case of the Latin American databases LILACS and SciELO, the following keywords and Boolean operators were used to search in Portuguese: (“muscular strength” OR “physical fitness”) AND (“bone mineral density” OR “bone mineral content” OR “bone health” OR “bone mass” OR “peak bone mass”). The authors and the list of references of the articles were additionally consulted.

The eligibility criteria were:

- Longitudinal studies.
- Containing the relationship between indicators of neuromuscular fitness in childhood and/or adolescence with variables of bone strength at least in adulthood within the objectives of the investigation.
- Not being a review or systematic review study.
- Not involving participants with pathologies, athletes, or animal models.

After the exclusion of studies according to the eligibility criteria, the full texts were evaluated and excluded if they did not use variables of bone strength, neuromuscular fitness with indicators only in adult age, presented a cross-sectional design, and did not meet the research objective.

It should be mentioned that despite the importance of having data on bone strength variables in early life and in adulthood to control genetic factors, tracking studies were also considered in the search due to the possibility that neuromuscular fitness in early life is used to predict bone health in adulthood.^21^ Selection and analysis of the studies were independently conducted by two researchers (C.C.L.B., C.L.P.R.) and, in case of divergence, a third researcher (E.R.V.R) was invited to decide whether to include or exclude the studies.

**Figure 1:**
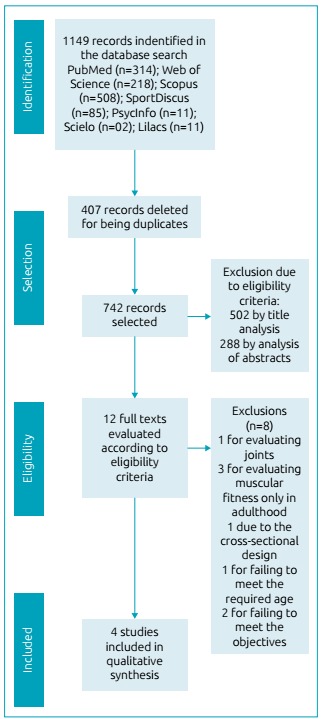
Diagram of the article selection process.

Additionally, an adaptation of the STROBE checklist was utilized,^22^ which is a commonly used tool to guide the reporting of observational studies, in order to evaluate the quality of the articles included in the study. The items selected and adapted for the present systematic review are presented in Table 1. The adaptation of the STROBE checklist for the quality classification of studies included in this systematic review consists of 15 items of analysis, with each accounting for a point or a half point when subdivided. Thus, if the article fits all the items, it receives a total score of 15 points. The cut-off points established for quality classification were: zero to five points=low quality; between six and 10 points=moderate quality; and 11 to 15 points=high quality. The studies that achieved moderate and high quality, according to the criteria mentioned above, were included in the final analysis. The process of selection and exclusion of articles is demonstrated in Figure 1.

**Table 1:** Adaptation of the STROBE checklist for quality classification of the studies.

Item	Topic and recommendation
1	Title and Abstract: a) Included in the title and/or abstract at least one of the keywords used in the search; b) Abstract provides a synopsis of what was performed and found.
2	Introduction: Explained the reasons and scientific basis for conducting the research.
3	Introduction: Includes general and specific objectives clearly considering any pre-established hypotheses.
4	Methods: Presented at the beginning of the document the key elements of the study design.
5	Methods: Described the relevant context, places, and dates, including recruitment, exposure, monitoring, and data collection periods.
6	Methods: Presented the eligibility criteria, as well as the sources and methods of selecting the participants. Specify follow-up methods when applicable (cohort studies).
7	Methods: Clearly defined all variables: response, exposure, predictive, confounding, and effect modifiers. If applicable, provided diagnostic criteria.
8	Methods: Specified and detailed the methods and instruments of measurement.
9	Methods: Sample size was rationally determined.
10	Methods: a) Explained how the quantitative variables were treated in the analysis; b) At least one statistical test was performed to verify the relationship in question.
11	Results: a) Presented at least one numerical information related to the analysis of the relationship in question; b) If variables categorized, described the cut-off points.
12	Results: Provided unadjusted estimates and, if applicable, adjusted for confounding factors (specify), as well as their accuracy (e.g., 95% confidence intervals).
13	Discussion: Summarized the main results of the study.
14	Discussion: Discuss the limitations, consider possible sources of bias or inaccuracy.
15	Discussion: a) Presented a prudent overall interpretation of the results considering the objectives, limitations, multiplicity of analyzes, and results of similar studies; b) Discussed the possibility of generalizing the results (external validity).

The studies were located in the databases consulted, filtered by language, and imported into *EndNote*. Initially, duplicate studies were excluded, and subsequently, studies were excluded after analysis of the titles, abstracts, or entire reading, according to the eligibility criteria. Evaluation of the quality of the included studies demonstrated that they were all considered to be “high quality” (Table 2).

**Table 2:** Description of the quality evaluation of studies included in this systematic review. Criteria adapted from the STROBE checklist.

References - online databases	Item															Total
	1	2	3	4	5	6	7	8	9	10	11	12	13	14	15	
1. Kemper et al., (2000)^18^	1	1	1	1	1	1	1	1	0.5	1	1	1	1	0	0.5	13
2. Delvaux et al., (2001)^23^	1	1	0.5	1	1	1	1	1	0.5	1	1	1	1	1	0.5	13.5
3. Barnekow-Bergkvist et al., (2006)^24^	1	1	1	0.5	1	1	1	1	0.5	1	1	1	1	1	1	14
4. Foley et al., (2008)^17^	1	1	1	0.5	1	1	1	1	1	1	1	1	1	1	0.5	14

## RESULTS

A synthesis of the main results of the studies is available in Table 3.

**Table 3:** Results of the studies included in the systematic review.

Author/ages	Sample	Neuromuscular fitness	Bone strength	Results
Kemper et al.^18^ BS: 13-16 years FUp: 21-27 years	83 Boys 98 Girls	Battery with 7 tests: ArmP, BAH, 10 LL, 10x5 m SR, S-R, plate tapping, SLJ; single score	BMD lumbar (L1-L4), femoral neck, distal radius; DXA	Regression coefficient revealed that neuromuscular fitness in adolescence was correlated with lumbar (0.16; p=0.05) and femoral neck BMD (0.18; p=0.05)
Delvaux et al.^23^ BS: 13 years FUp1: 18 years FUp2: 40 years	126 Boys	LL, BAH, vertical jump, ArmP; Absolute score of change in performance was calculated between 18 and 13 years	BMD and BMC TB and lumbar region; DXA	Correlated in ArmP and the TB (r=0.19*) and lumbar BMC (r=0.21*), and in LL and the BMC of the TB (r=0.19*). At 18 years, BAH with total BMC (r=0.21*), ArmP total BMC (r=0.28*) and lumbar BMC (r=0.27*)
Barnekow-Bergkvist et al.^24^ BS: 15-17 years FUp: 35-37 years	36 Girls	Hanging LL, handgrip, two HL	TB, arms, legs, lumbar, trochanter, femoral neck BMD; DXA	Two HL was predictor for TB BMD (R^2^adj=0.10*); the hanging LL (R^2^adj=0.12*) and handgrip (R^2^adj=0.08*) for BMD arms; and the two HL for BMD legs (R^2^adj=0.11*) and BMD trochanter (R^2^adj=0.08*)
Foley et al.^17^ BS: 7-15 years FUp: 26-36 years	691 Boys 743 Girls	Leg strength test with dynamometer, SLJ	Ultrasound of calcaneal	In boys, no measure was predictive of quantitative ultrasound parameters. In girls too, after adjusted for performance adult, SLJ with QUI (β=0.07) and with speed of sound (β=0.08)

BS: baseline; FUp: follow-up; ArmP: arm pull; BAH: bent arm hang; LL: leg lift; SR: shuttle run; S-R: sit and reach; SLJ: standing long jump; HL: hand lift; BMD: bone mineral density (g/cm^2^); BMC: bone mineral content (g); TB: total body; DXA: Dual-energy X-ray absorptiometry; QUI: quantitative ultrasound index; *p-value<0.05 or p-value<0.01.

### Characteristics of the studies

The majority of the studies were carried out in European countries, with baseline collections in the 70s and follow-ups of 15 to 27 years.^18^ ^,^ ^23^ ^,^ ^24^ One study was performed in Australia in 1985 about a 20-year follow-up.^17^ In total, four different samples were followed, with data from the Amsterdam Growth and Health Longitudinal Study,^18^ Leuven Longitudinal Study on Lifestyle, Physical Fitness and Health,^23^ Swedish students,^24^ and Australian Schools Health and Fitness Survey.^17^

Regarding the characteristics of studies, only two involved one sex,^23^ ^,^ ^24^ and two studies involved both sexes.^17^ ^,^ ^18^ In these studies, sex control was adopted (sex-adjusted analysis^18^ and stratified analysis)^17^ because it was a determinant of bone strength. The studies were published between 2000 and 2008. The most recent has the largest sample and covered childhood.^17^

### Methods used to obtain the bone strength variables

The study by Foley et al.^17^ used calcaneal ultrasound to evaluate bone strength parameters, consisting of speed of sound (SOS, m/s), broadband ultrasound attenuation (BUA, dB/MHz), and quantitative ultrasound index (QUI) using the equation QUI = 0.41 x (BUA + SOS) - 571.

In all other studies, dual-energy X-ray absorptiometry (DXA) was used to obtain BMC and/or BMD, although different equipment was used (Hologic, Norland, Lunar). Different bone sites were measured, such as whole body, lumbar spine, femoral neck, distal radius, whole arms and legs, and greater trochanter. Evaluating different bone sites allows the analysis of associations of anatomical specificities about predominant musculature involved in the test applied as an indicator of neuromuscular fitness.

All studies obtained the information on bone strength variables only in the follow-up period, that is, in the adult phase. The measurement of the whole body with DXA involved the head, since the recommendation to exclude the head is related only to children and adolescents.^25^

### Methods used to obtain indicators of neuromuscular fitness

Neuromuscular fitness was measured in four studies by using motor tests to obtain indicators of different physical capacities: standing long jump (explosive leg strength);^17^ leg lifts and sit-ups (trunk muscle strength), bent arm hang (upper body strength), vertical jump (explosive leg strength), arm pull (static arm strength);^23^ battery neuromotor fitness - arm pull, bent arm hang, 10 leg lifts (trunk/leg strength), 10x5m shuttle run (speed and agility), sit and reach (trunk flexibility), plate tapping (eye-hand coordination and arm speed), standing high jump;^18^ hanging leg lift (muscle strength).^24^ In two studies, dynamometry was also used to evaluate muscle strength: handgrip, two-hand lift,^24^ and leg strength tests.^17^ Predominance of tests that evaluate the physical capacities of muscular and explosive strength, muscular actions that provide great tension in bones was observed.

While Kemper et al.^18^ used a battery of motor tests and unified the performance in the seven tests into a general score, the other studies analyzed the performance in each test of neuromuscular fitness and its association with variables of bone strength.^17^ ^,^ ^23^ ^,^ ^24^ In the study by Barnekow-Bergkvist et al.,^24^ neuromuscular fitness was only evaluated at baseline, while in the other studies evaluation was performed at both collection moments.^17^ ^,^ ^18^ ^,^ ^23^

### Associations between neuromuscular fitness and bone strength

A study that covered the period of childhood at baseline identified in females a positive and significant relationship between standing long jump with QUI (β=0.11; p<0.05) and SOS (β=0.14; p<0.01), when adjusting the model by childhood age and body mass index (Z score). However, when the model included jump performance in adulthood, there was attenuation of the magnitude and loss of significance in the association (β=0.07; β=0.08, respectively).^17^ That is, at first, girls with better jump perfomance had a better indicator of bone strength. However, it seems to be necessary to maintain a good performance in the jump in adulthood, since the relationship ceased to exist when controlling for this variable.

Studies involving the adolescence period at the baseline demonstrated that, for both sexes, higher performance in adolescent neuromuscular fitness correlated with bone strength in adulthood,^18^ ^,^ ^23^ ^,^ ^24^ with coefficients varying from 0.16 (neuromotor battery and lumbar BMD) to 0.38 (hanging leg lift test with BMD arms).^18^ ^,^ ^24^

When analyzing the magnitude of the relationship between variables, a discrete variation was observed between studies, enough to classify the correlation as weak and moderate,^18^ ^,^ ^23^ such as the correlations that were found between performance in the two-hand lift test and the total body and legs BMD (r=0.33‒0.35), and between the hanging leg lift with arms BMD (0.38).^24^ Explained variance varied from 2%, as in the bent arm hang at age 18 for BMD and total BMC, 3% as in the leg lifts at age 13 for total and lumbar BMC,^23^ to 12%, as in the hanging leg-lift at 16 for BMD arms.^24^ Despite these discrete percentage values, neuromuscular fitness may be an important factor, in view of the variety of many other factors that determine optimization of peak bone mass.

Concerning bone sites, from the regions of interest, no significant associations were found with indicators of neuromuscular fitness in the distal radius,^18^ the lumbar region and femoral neck in one of the studies,^24^ and the calcaneal region measured by ultrasound.^17^ It should be highlighted that there was no standardization regarding the type of test applied and bone sites measured, making it difficult to interpret and compare the results from different experiments.

## DISCUSSION

In adults, bone strength seemed significantly and directly correlated in low to moderate magnitude with indicators of neuromuscular fitness in adolescence. In the study involving childhood in the baseline, the relationship was not found.

The literature is lacking regarding systematic review about this targeted relationship, as neuromuscular fitness in childhood and/or adolescence is a determinant of the peak bone mass. Despite the number of cross-sectional studies, the literature investigating the contribution of neuromuscular fitness to bone strength variables,^15^ ^,^ ^16^ ^,^ ^26^ ^,^ ^27^ the number of longitudinal observational investigations that examine this interaction from young people to adulthood is still limited. This was evident since, through this systematic review of the literature, only four studies of this nature investigating this phenomenon were found.^17^ ^,^ ^18^ ^,^ ^23^ ^,^ ^24^

### Characteristics of the studies

Developing longitudinal studies is very complex. Among the challenges, the maintenance of the number of individuals over the years is highlighted, which may explain the difficulty in finding studies from childhood. This dropout effect must be observed because it can influence the generalization of the results. Approximately 17% of the baseline sample participated in the follow-up studies by Foley et al.^17^ and Barnekow-Bergkvist et al.^24^ In both studies, a dropout effect was observed, with a small magnitude in the first, although with a possible compromise to the external validity and the significance of some relationships in the second, due to the smaller sample size. Delvaux et al.,^23^ who began the study with 441 eligible boys and concluded with 126, did not report dropout information. Kemper et al.,^18^ who evaluated 307 participants initially and after 15 years assessed 182, was not affected by dropouts.

The studies controlled in their analyzes variables such as sex, chronological and biological ages, and anthropometry and body composition because these variables may influence bone mass. In both sexes, significant positive relationships were found when the baseline was adolescence, with coefficientes of greater magnitude for girls.^24^ Faulkner et al.^28^ also found advantages for girls (r=0.51) when compared to boys (r=0.13) in the association between physical fitness in adolescence and adult BMD. Foley et al.^17^ suggest that the effects of exercise during childhood on adult bone mass lead to more long-term benefits in females. Different effects of physical activity during adolescence on bone strength, content, and size were observed by Duckham et al.,^29^ who speculated that these responses arose from bone formation, hormonal increases in adolescence, and the type and intensity of physical activities inherent to sex.

Regarding age control, most of them presented homogeneous chronological age, ^18^ ^,^ ^23^ ^,^ ^24^ and regarding biological age, one study considered the age at menarche,^24^ another study estimated skeletal age,^18^ and two studies did not present maturation information.^17^ ^,^ ^23^ Exposure to mechanical loads during childhood is reinforced by Gunter et al.^30^ for optimization of skeletal health throughout life; therefore, the relationship between neuromuscular fitness and bone strength needs to be verified as early as possible (childhood). The fact the study that evaluated childhood in the baseline did not found find a relationship between the variables when considering the adult performance in the jump might suggest that stimuli that promote better muscle performance need to be maintained throughout life for the preservation of the osteogenic effects.^17^

Although the bone tissue constantly adapts to the stimuli received, it is during the period of physical growth that bones respond better to mechanical loading stimuli.^19^ ^,^ ^31^ ^,^ ^32^ Baxter-Jones et al.^8^ observed that 39% of the total body BMC was attained within ±2.5 years of peak height velocity (PHV) and that peak bone mass gain occurred about one year after PHV, for both sexes. The positive relationships of the studies referring to adolescence period at baseline may indicate neuromuscular fitness as an indirect parameter of monitoring the acquisition of bone mass during the years of physical growth.

As many factors can influence peak bone mass,^5^ ^,^ ^6^ the studies found in this review also considered other variables. Concerning anthropometry and body composition, the models were adjusted for height, body mass and sum of skinfolds,^18^ body mass index,^17^ ^,^ ^23^ and alteration in body mass between follow-up and baseline.^24^ Other variables considered were information related to nutrition,^18^ ^,^ ^23^ ^,^ ^24^ smoking habit in adulthood,^23^ ^,^ ^24^ and the use of oral contraceptives.^24^

### Methods used to obtain indicators of neuromuscular fitness and bone strength variables

Different methods were used to obtain the variables, making it difficult to compare the studies. For neuromuscular fitness, methods ranging from dynamometers and motor tests analyzed separately to the neuromotor battery, in which a score resulted from different neuromotor components (57% involving muscle strength), which may have influenced the relation.^18^ From the motor tests, those indicators of muscle strength were those that presented significant relationships.^23^ ^,^ ^24^ Strength, endurance, and muscle power may be distinctly related to the variables of bone strength, possibly due to the tension in the bones according to the type of muscular action, a specificity which can be observed in the studies.^14^ ^,^ ^15^ ^,^ ^17^ ^,^ ^23^

It is also interesting that the musculature predominantly involved in the test is close to the measured bone site and whether or not it involves the support of the body mass. Kemper et al.,^18^ for example, found no statistically significant association between physical fitness and distal radius BMD. In addition to the biological individuality, each bone needs a deformation threshold to present a reaction and, possibly, there is also a certain response according to the type of bone (trabecular, cortical) predominant in the region, leading to the importance of measuring the greatest possible number of sites. Barnekow-Bergkvist et al.^24^ measured total body, lower and upper limbs, lumbar region, and femur, making it possible to relate the measurements with neuromuscular fitness tests with implications in specific body regions.

In the only study using ultrasound, only the calcaneal region was evaluated, however neuromuscular fitness indicators that observed site specificity were used.^17^ Parameters of bone ultrasonometry are associated with fracture risk,^33^ however, although other techniques are interesting in the analysis of the bone status and fracture risk, the majority of studies of peak bone mass have used DXA,^6^ an extremely precise method to quantify BMD and body composition.^34^

### Associations between neuromuscular fitness and bone strength

The magnitude of the coefficients of the studies was low to moderate. One of the factors that may interfere in the magnitude of the relationship between neuromuscular fitness indicators and bone strength variables is the period between measurements, that is, as the time interval increases, the coefficients tend to decrease. For example, significant and higher magnitude coefficients are observed when the relationships are investigated in the same phase of life, as adulthood vs. adulthood, when compared from one phase to another in life, such as adolescence vs. adulthood.^23^ The only study involving childhood neuromuscular fitness and bone strength variables in adults did not demonstrate significant relationships after controlling for adult performance.^17^

This loss of significance after control for adult performance suggests that muscular fitness is an important determinant if sustained from childhood to adulthood.^17^ While bone strength will increase through the acquisition of bone in stimulated regions, in the absence of stimulation, bone mass will be removed,^7^ demonstrating a constant adaptation of bone to mechanical loads throughout life. Of the studies included in this systematic review, only one did not assess adult performance in neuromuscular fitness.^24^

It is not yet clear in the literature how much the benefits obtained during youth can be maintained in adulthood. A study of physically active men and women in adolescence presented BMC, in total body and hip regions, 8 to 10% greater than their inactive or moderately active pairs. It should be mentioned that a higher physical activity score was found among adults classified as physically active during adolescence.^35^ In studies involving athletes, there are discrepancies regarding the advantages in adults of exposure to mechanical loads in childhood/adolescence when the stimulus is reduced or suspended in adulthood.^36^ ^,^ ^37^ ^,^ ^38^

Studies on the relationship between neuromuscular fitness and bone strength have considered the mediating effect of other variables. A study involving children showed that the relationship between indicators of neuromuscular fitness and bone parameters was totally mediated by lean mass.^39^ A study with adolescents demonstrated no independent relationship between physical fitness (EUROFIT battery) and total BMC body after the models were adjusted for lean mass, but rather an independent relation between lean mass and bone mass, explaining 67% of the total independent variation in age and sex.^40^ No studies were found that investigated mediating effect when analyzed the relationship of neuromuscular fitness in childhood/adolescence and bone strength in adulthood.

Thus, there is limited evidence (reduced number of studies, heterogeneity of applied tests and bone sites, and dropout effect) to support the neuromuscular fitness in early life as a determinant of bone strength in adulthood. The fact that the magnitude of the relationship is low to moderate does not diminish the importance of stimulating the improvement of neuromuscular fitness in childhood and adolescence, given the sensitivity of the skeleton to mechanical loads during the first two decades of life and the increase in bone mass during growth years being essential for bone strength, postponement of the onset of osteoporosis, and reduction in the risk of fractures.

Based on the above, the following are suggested: prospective studies with large sample size; the follow-up of male and female strata for several years starting at early ages such as childhood; the measurement of several bone sites and the application of tests indicating neuromuscular fitness considering site specificities at multiple moments of the follow-up; the control of neuromuscular fitness performance levels in adulthood, as well as other variables that influence bone mass; analysis of the impact of the performance trajectory on neuromuscular fitness in bone mass, besides the potential effect of biological maturation during the youth period.

## CONCLUSIONS

Neuromuscular fitness in childhood demonstrated a statistically significant positive relationship with variables of bone strength at adult age in females, and this relationship was not maintained after control for neuromuscular performance in adulthood. Neuromuscular fitness in adolescence seems to be related positively, low to moderate magnitude, with bone strength in adulthood. However, there is limited evidence to support the neuromuscular fitness in early life as a determinant of bone strength in adulthood.
